# Comparison of bleeding risk scores and evaluation of major bleeding predictive factors in patients with major bleeding due to vitamin K antagonist use

**DOI:** 10.1016/j.heliyon.2023.e19079

**Published:** 2023-08-12

**Authors:** Sinan Yildirim, Onur Aslan

**Affiliations:** a Department of Emergency Medicine, Canakkale Mehmet Akif Ersoy State Hospital, Canakkale, Turkey; b Department of Cardiology, Tarsus State Hospital, Mersin, Turkey

**Keywords:** Atrial fibrillation, Vitamin K antagonist, Major bleeding, GARFIELD-AF, HAS-BLED

## Abstract

**Background:**

Major bleeding in the treatment of atrial fibrillation is closely associated with an increased risk of death and major adverse outcomes in both the short and long term, but all bleeding events are associated with a reduced quality of life. Bleeding events are also known to reduce medication adherence. In this sense, bleeding risk scores are important tools to help predict major bleeding. However, it is not clear which scoring system is superior.

**Aim:**

In this study, our aim was to compare bleeding risk scores and to examine the factors associated with bleeding in patients with major bleeding while using vitamin K antagonists.

**Methods:**

In this retrospective and single-center study, scoring, laboratory and demographic data were analyzed with SPSS 20.0 statistical program.

**Results:**

The mean age of a total of 1434 patients included in our study was 68.2 ± 11.3 years, range was 39–93 years and 769 (53.6%) of these patients were male. Of 588 patients with major bleeding, 93 (15.8%) had intracranial hemorrhage. Logistic regression analysis comparing the scoring systems among themselves revealed that the GARFIELD-AF scoring system had a predictive effect on major bleeding independent of the effect of other scoring systems (OR: 1.532, 95% CI 1.348–1.741, p < 0.001). The area under the curve (AUC) for GARFIELD-AF was 0.690 (0.662–0.718) as a result of the ROC analysis considering the best cut-off point of 3.2% calculated for 2 years. AUC 0.659 (0.630–0.687) for HAS-BLED, AUC 0.636 (0.606–0.665) for ORBIT and AUC 0.611 (0.5810.642) for ATRIA. When we compare the patient group with the control group, it can be said that intracranial hemorrhage occurred independently of INR and TTR values, unlike in the major bleeding group (p:0.129, p:0.545).

**Conclusion:**

In patients using vitamin K antagonists for atrial fibrillation, the GARFIELD-AF risk score was found to be superior to important bleeding risk scores such as HAS-BLED, ORBIT and ATRIA in terms of predicting major bleeding. It is an important result that intracranial hemorrhages, which have a special place among major hemorrhages, were independent of INR and TTR levels. It is noteworthy that 8.2% of patients with major bleeding had a history of minor bleeding in the last year.

## 1 Introduction

Atrial fibrillation (AF) is the most common cardiac arrhythmia and a well-recognized cardiac pathology with a clear association with stroke and death. The five-fold increase it causes in stroke risk is the condition that directs the treatment of AF [1]. With stroke being the biggest risk, the impact on quality of life should not be overlooked. Its significantly increasing incidence with age, together with increasing life expectancy, has led to its increasing prominence, especially in cardiology and neurology clinics [2]. Treatment designed to prevent stroke is closely associated with increased risk of bleeding [3].

In order to establish this balance in the best way, there are stroke-related scoring systems such as CHA2DS2-VASc [4], as well as scoring systems assessing bleeding risk such as HAS-BLED, GARFIELD-AF, ATRIA, ORBIT [5,6]. HAS-BLED, an effective scoring system, has found an important place in clinical practice. HAS-BLED score is originated from the European Heart Survey database in 2010 [1], mainly focusing on the modifiable bleeding risk factors. The HAS-BLED score has been routinely recommended for predicting the bleeding risks in patients with AF who use anticoagulant [4,7]. However, many bleeding prediction scoring systems have been put forward [1,5,8]. It is unclear which one is the best and has the most practical ease of use. While there are studies indicating the superiority of the HAS-BLED score [[9], [10], [11]], there are also studies reporting contrary results [5,12].

At this point, it should be noted that no matter which scoring system is used, although the aim of these systems is to assess the risk of bleeding during treatment, a high bleeding risk score is not an excuse to withhold oral anticoagulants (OAC) [3].

Major bleeding has been evaluated as a safety endpoint in OAC studies and in studies comparing warfarin with non-vitamin K antagonist oral anticoagulant (NOAC) drugs such as rivaroxaban, apixaban and edoxaban. Major bleeding rates were found to be 3.4%, 3.1% and 3.4% for warfarin, respectively [[13], [14], [15]].

With the distinction between major and minor bleeding complications, intracranial hemorrhages have a much more special place in terms of morbidity and mortality among major hemorrhages. Intracranial hemorrhages, which have an annual incidence of 0.2%, are reported to be around 0.5% in major NOAC studies, whereas this figure may be as high as 0.9% in patients using Vitamin K Antagonists (VKA) [[14], [15], [16]].

In other words, when practicing the art of medicine between stroke prevention therapy and bleeding risk, clinicians must also face the clear scientific recommendation that the primary goal is stroke prevention and that bleeding risk should not be determinative for the treatment. It has been emphasized many times that high bleeding risk should not be a determinant in anticoagulant prescription [3,4]. Despite all these, bleeding complications are the most important source of morbidity and mortality for both the patient and the physician. Bleeding scores used for this purpose have been frequently questioned in the literature. Although HAS-BLED scoring has been compared with other scoring systems many times and significant results have been demonstrated in favor of HAS-BLED [[17], [18], [19]], there are also studies that prevent a clear superiority [5,8,12].

Our aim in this study was to compare bleeding risk scores and to examine factors associated with bleeding in patients who had major bleeding while on VKA use.

## 2 Methods

### 2.1 Study setting

The study was conducted as a single-center study among patients with major bleeding due to VKA use who presented to the emergency department between January 1, 2009 and January 1, 2013 and control group patients who came for measurement of international normalized ratio (INR) and did not have any bleeding.

### 2.2 Study population and data collection

Our study was conducted in the emergency department of a tertiary education and research hospital with a daily average of 1200–1400 patients. We retrospectively reviewed 4 years of data through the hospital information system. Hospital admissions of patients with AF who used VKA were analyzed. The files of the patients admitted to the emergency department with major bleeding and those of the control group were retrieved from the archival records. Records not included in the data in the information system were recorded through patient files. Anamnesis, examination results, laboratory data, clinical follow-up and treatment of the patients were filled in a form. Patients' medication reports were obtained from the pharmacy registration system. Patients with missing anamnesis information were contacted through their registered phones to complete the data.

Patients who used VKA for reasons other than atrial fibrillation, who presented with trauma, who were not regularly monitored for INR, who had a hematologic disease that could cause bleeding, who presented with clinically related non-major or minor bleeding outside the criteria for major bleeding, and whose hospital data could not be fully accessed were excluded from the study. All other patients who did not have major bleeding and met the inclusion criteria were included as the control group in order not to disrupt randomization in patients who came to the emergency department. A total of 1434 patients, 588 patients with major bleeding and 846 controls, were included in the study.

International Society on Thrombosis and Haemostasis (ISTH) major bleeding criteria were used as major bleeding criteria [20]:

• Fatal bleeding, and/or
• Symptomatic bleeding in a critical area or organ, such as intracranial, intraspinal, intraocular, retroperitoneal, intra-articular or pericardial, or intramuscular with compartment syndrome, and/or
• Bleeding that causes a decrease in hemoglobin level of 2 g/dL (1.24 mmol/L) or more or requires two or more units of whole blood transfusion

The following criteria were used for Clinically Relevant Non-Major bleeding (CRNM) [20].

• Requiring medical intervention by a healthcare professional
• Leading to hospitalization or increased level of care
• Prompting a face to face (i.e., not just a telephone or digital communication) evaluation

History of hypertension, transient ischemic attack (TIA) or stroke, vascular disease, chronic kidney disease (CKD), congestive heart failure (CHF), diabetes mellitus (DM), malignancy and anemia were recorded on the form. Liver and kidney function values were processed according to the laboratory tests performed before the emergency department admission due to hemorrhage. INR values measured in the one-year period before the emergency department visit were recorded. Patients with any evidence of acute renal or liver failure at presentation were excluded. The history of major and minor bleeding of the patients in the last one year was obtained. Major bleeding and bleeding other than CRNM were considered minor bleeding. Information on the medication used by the patients was accessed from the pharmacy registration system with their ID numbers.

The risk scores HAS-BLED (Hypertension, Abnormal liver/renal function, Stroke, Bleeding, Labile International Normalized Ratio, Elderly, Drugs or alcohol), ORBIT (Older age, Reduced haemoglobin/haematocrit/anaemia, Bleeding history, Insufficient kidney function, Treatment with platelets), ATRIA (Anticoagulation and Risk Factors in Atrial Fibrillation) and GARFIELD-AF (Global Anticoagulant Registry in the FIELD-Atrial Fibrillation) were used.

GARFIELD-AF bleeding score was calculated at 2 years. Asia was marked as the race of choice in our patients. The diastolic blood pressure, weight, and pulse rate parameters in the scoring were the average of the values obtained at the time of admission and at admission within the last 1 year.

Time in therapeutic range (TTR) was calculated by the Rosendaal method and INR values measured during the last 1 year were noted [21].

### 2.3 Statistical analyses

SPSS 20.0 for Windows® statistical program (IBM Inc. Chicago, IL, USA) was used. Number, percentage, mean, standard deviation, median, minimum and maximum were used to present descriptive data. The conformity of the data to normal distribution was evaluated by Kolmogorov-Smirnov Test. Pearson chi-square test and Fisher's Exact test were used to compare categorical data. T Test was used for the comparison of two independent numerical data and Mann Whitney u test was used when the data were not equally distributed, Kruskal Walles Test and Analysis of variance (ANOVA) test were used for the comparison of three independent numerical data. The evaluation of risk scores among themselves was evaluated by binary logistic regression analysis. Receiver operating characteristic (ROC) analysis was performed for all risk factors. With this analysis, area under the curve, cut-off values, sensitivity and specificity values were found.

Results were considered significant at p < 0.05, with a 95% confidence interval.

### 2.4 Ethical considerations

Ethics committee approval was obtained from the ethics committee of the Ministry of Health, Dışkapı Yıldırım Beyazıt Training and Research Hospital (December 17, 2012, 06/36).

The entire study was conducted in accordance with the Declaration of Helsinki.

## 3 Results

The mean age of a total of 1434 patients included in our study was 68.2 ± 11.3 years, range was 39–93 years and 769 (53.6%) of these patients were male. Of 588 patients with major bleeding, 93 (15.8%) had intracranial hemorrhage.

The gender distribution of the patients with major bleeding and the control group was similar (p:0.568). In terms of age groups, the proportion of patients over 65 and over 75 years of age was higher in patients with major bleeding compared to the control group (p:0.021, p < 0.001, respectively). There was no significant difference between the mean weight and pulse rate (p:0.205, p:0.087, respectively). Labil INR, liver enzymes and renal function markers were significantly higher in the group of patients with major bleeding (p < 0.001). There was no difference between both groups in terms of non-steroidal anti-inflammatory drug use and smoking (p:0.256, p:0.105, respectively). Antiplatelet agent and alcohol use were higher in patients with major bleeding (p < 0.001, p:0.003, respectively). Diastolic blood pressure, hypertension, malignancy, history of previous minor and major bleeding were significantly higher in patients presenting with major bleeding compared to the control group (all p < 0.001). There was no difference between the two groups in terms of history of stroke, vascular disease and anemia (p:0.127, p:0.155, p:0.289, respectively). When INR and TTR values of both groups were compared, statistically significant difference was observed (both p < 0.001) (Table 1).

**Table 1:** Comparison of patients who developed major bleeding in patients with atrial fibrillation who used VKA with the diagnosis of atrial fibrillation with the control group in terms of risk factors.

Characteristics	Total (n:1434)	Major bleeding		p value
		Yes (n:588)	No (846)	
Age groups				
>65	796 (55.5%)	365 (62.1%)	431 (51.0%)	**0.021**
>75	547 (38.2%)	276 (46.9%)	271 (32.0%)	**<0.001**
Female	665 (46.4%)	276 (46.9%)	389 (46.0%)	0.568
Weight (kg)	76.7 ± 13.1	79.6 ± 14.6	74.6 ± 12.1	0.205
Pulse (bpm)	82.5 ± 18.9	86.6 ± 19.8	79.6 ± 18.2	0.087
Diastolic blood pressure (mmHg)	86.2 ± 15.3	96.3 ± 19.2	79.1 ± 12.7	**<0.001**
Hypertension	703 (49.0%)	365 (62.1%)	338 (40.0%)	**<0.001**
Previous stroke/TIA	152 (10.6%)	76 (12.9%)	76 (9.0%)	0.127
Heart Failure	368 (25.7%)	182 (31.0%)	186 (22.0%)	**0.001**
Diabetes	258 (18.0%)	123 (20.9%)	135 (16.0%)	0.056
Vascular disease	287 (20.0%)	135 (23.0%)	152 (18.0%)	0.155
Malignancy	130 (9.1%)	71 (12.1%)	59 (7.0%)	**<0.001**
Abnormal liver function	20 (1.4%)	12 (2.0%)	8 (1.0%)	**<0.001**
Stage III or IV chronic kidney disease	87 (6.1%)	53 (9.0%)	34 (4.0%)	**<0.001**
Anemia	233 (16.3%)	106 (18.0%)	127 (15.0%)	0.289
Alcoholism	20 (1.4%)	12 (2.0%)	8 (1.0%)	**0.003**
Antiplatelet use	116 (8.1%)	65 (11.1%)	51 (6.0%)	**<0.001**
NSAIDs use	106 (7.4%)	47 (8.0%)	59 (7.0%)	0.256
Current smoker	472 (32.9%)	209 (35.5%)	263 (31.1%)	0.105
Previous major bleeding	344 (24.0%)	200 (34.0%)	144 (17.0%)	**<0.001**
Previous minor bleeding	32 (2.2%)	48 (8.2%)	8 (1.0%)	**<0.001**
Labile INR	183 (12.8%)	99 (16.8%)	84 (9.9%)	**<0.001**
**INR**				
2<	59 (4.1%)	12 (2.0%)	47 (5.6%)	**<0.001**
2–3	675 (47.1%)	143 (24.3%)	532 (62.9%)	
3>	700 (48.8%)	433 (73.6%)	267 (31.6%)	
**TTR**				
50<	476 (33.2%)	245 (41.7%)	231 (27.3%)	**<0.001**
50–60	658 (45.9%)	261 (44.4%)	397 (46.9%)	
60>	300 (20.9%)	82 (14.0%)	218 (25.8%)	
**Risk Scores in Predicting**				
HAS-BLED		3.3 ± 1.6	2.4 ± 1.3	**<0.001**
ORBIT		4.0 ± 1.6	3.3 ± 1.4	**<0.001**
ATRIA		4.8 ± 1.9	4.2 ± 1.6	**<0.001**
GARFIELD-AF		3.4 ± 1.9	2.2 ± 1.2	**<0.001**

**TIA**, transient ischaemic attack; **NSAID**, non-steroidal anti-inflammatory drug; **INR**, international normalized ratio; **TTR**, time in this therapeutic range; **HAS-BLED**, Hypertension, Abnormal liver/renal function, Stroke, Bleeding, Labile International Normalized Ratio, Elderly, Drugs or alcohol; **ORBIT**, Older age, Reduced haemoglobin/haematocrit/anaemia, Bleeding history, Insufficient kidney function, Treatment with platelets; **ATRIA**, Anticoagulation and Risk Factors in Atrial Fibrillation; **GARFIELD-AF**, Global Anticoagulant Registry in the FIELD-Atrial Fibrillation;

When HAS-BLED, ORBIT, ATRIA and GARFIELD-AF scores were compared, it was observed that all scores were significantly higher in the patient group presenting with major bleeding (p < 0.001 for all scores) (Table 1).

Logistic regression analysis comparing the scoring systems among themselves revealed that the GARFIELD-AF scoring system had a predictive effect on major bleeding independent of the effect of other scoring systems (OR: 1.532, 95% CI 1.348–1.741, p < 0.001) (Table 2).

**Table 2:** Comparision of risk scores with logistic regression analysis.

	Cut-off	P Value	Odds Ratio	95% CI	
				Lower	Upper
HAS-BLED	3.5	0.373	1.062	0.931	1.211
ORBIT	4.5	0.356	1.049	0.948	1.161
ATRIA	5.5	0.296	1.043	0.964	1.129
GARFIELD-AF	3.2	**<0.001**	1.532	1.348	1.742

**HAS-BLED**, Hypertension, Abnormal liver/renal function, Stroke, Bleeding, Labile International Normalized Ratio, Elderly, Drugs or alcohol; **ORBIT**, Older age, Reduced haemoglobin/haematocrit/anaemia, Bleeding history, Insufficient kidney function, Treatment with platelets; **ATRIA**, Anticoagulation and Risk Factors in Atrial Fibrillation; **GARFIELD-AF**, Global Anticoagulant Registry in the FIELD-Atrial Fibrillation; **CI**, Confidence Interval.

**Fig. 1:**
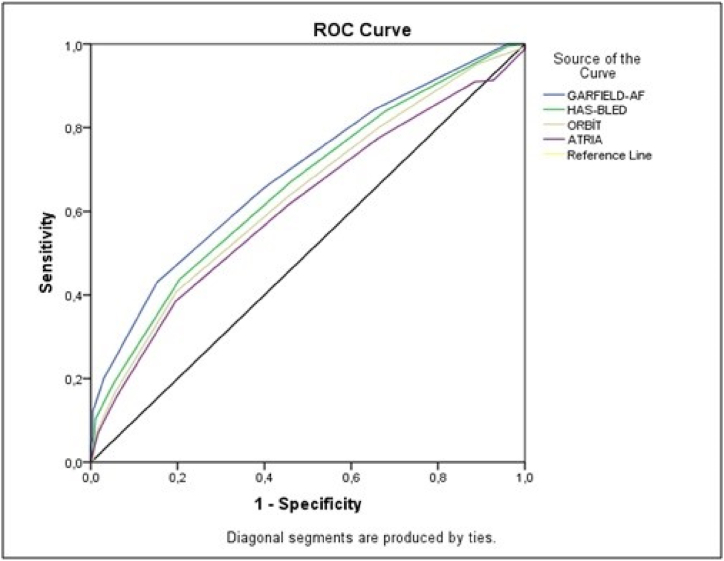
ROC analysis comparing scoring.

The area under the curve (AUC) for GARFIELD-AF was 0.690 (p < 0.001) as a result of the ROC analysis considering the best cut-off point of 3.2% calculated for 2 years. AUC 0.659 (p:0.008) for HAS-BLED, AUC 0.636 (p:0.017) for ORBIT and AUC 0.611 (p:0.022) for ATRIA (Table 3 and Fig. 1).

**Table 3:** Comparision of risk scores with ROC analysis.

	Cut-off	P Value	Area Under the Curve	Sensitivity	Specificity	95% CI	
						Lower	Upper
HAS-BLED	3.5	**0.008**	0.659	43.7	79.6	0.630	0.687
ORBIT	4.5	**0.017**	0.636	40.8	80.3	0.606	0.665
ATRIA	5.5	**0.022**	0.611	38.4	80.5	0.581	0.642
GARFIELD-AF	3.2	**<0.001**	0.690	43.0	84.8	0.662	0.718

**HAS-BLED**, Hypertension, Abnormal liver/renal function, Stroke, Bleeding, Labile International Normalized Ratio, Elderly, Drugs or alcohol; **ORBIT**, Older age, Reduced haemoglobin/haematocrit/anaemia, Bleeding history, Insufficient kidney function, Treatment with platelets; **ATRIA**, Anticoagulation and Risk Factors in Atrial Fibrillation; **GARFIELD-AF**, Global Anticoagulant Registry in the FIELD-Atrial Fibrillation; **CI**, Confidence Interval.

ROC analysis results of HAS-BLED, ORBIT, ATRIA and GARFIELD-AF scores sensitivity and specificity values according to cut-off values are given in Table 2. GARFIELD-AF scoring was superior to other scoring systems in indicating major bleeding with 43.0% sensitivity and 84.8% specificity according to a cut-off value of 3.2% (Table 3 and Fig. 1).

**Fig. 2:**
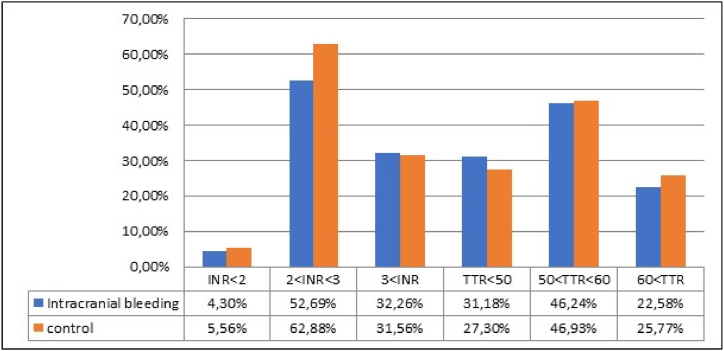
Comparison of INR and TTR values between intracranial hemorrhage and control group.

INR value was below 2 in four (4.3%), between 2 and 3 in forty-nine (52.7%) and above three in thirty (32.3%) patients with intracranial hemorrhage. TTR value was less than 50% in twenty-nine (31.2%) patients, between 50 and 60% in forty-three (46.2%) patients and 60% and above in twenty-one (22.6%) patients. When we compare the patient group with the control group, it can be said that intracranial hemorrhage occurred independently of INR and TTR values (p:0.129, p:0.545, respectively), unlike in the major bleeding group (Fig. 2).

## 4 Discussion

Our important findings in this study are; firstly, the predictive power of GARFIELD-AF risk scoring for major bleeding in patients receiving VKA for AF was found to be higher than HAS-BLED and some other scoring systems. Our second important result is that intracranial hemorrhages are seen independently of INR and TTR values in this group of patients using VKA. Our third main result is that a significant proportion (8.2%) of patients with major bleeding while using VKA, had a history of minor bleeding within one year.

Major bleeding is undoubtedly the leading cause of clinician concern about bleeding in the management of AF [22]. Major bleeding events are closely associated with an increased risk of death and major adverse outcomes in both the short and long term [6,23], but all bleeding events are associated with a reduced quality of life [24]. It is also known that bleeding events decrease drug compliance [25]. Although scoring such as HAS-BLED is increasingly emphasized in this regard, whether it is used as much as it should be is a question that has no clear answer, especially for busy clinics. At this point, risk scoring systems such as GARFIELD-AF, ATRIA, ORBIT are used with criteria similar to HAS-BLED at some points and differing at others.

In a study, simple obtainable parameters such as heart rate, diastolic blood pressure, and weight in the GARFIELD-AF risk scoring system seem to have made a difference from the analysis with a significant number of patients such as 52080. Another important point is that the bleeding risk in low-risk patients, who have very limited data in the literature, was also evaluated in this study, and the result remained significant [5]. The predictive power of a treatment prescribed for a very important benefit such as prevention of stroke in clinical practice, but without symptomatic effect, of a complication such as bleeding in low-risk patients with low morbidity, which may both cause serious mortality and morbidity in the patient and significantly affect the physician-patient relationship. We think it's important. Regarding bleeding, it has been shown to be superior to the HAS-BLED score, especially in the low-risk patient profil. In another important study, this superiority was validated for CHA2DS2-VASc in ischemic stroke and evaluated as comparable to HAS-BLED in major bleeding [12]. In a meta-analysis conducted by Gao X et al. including patients using NOAC and VKA, it was concluded that GARFIELD-AF risk scoring was noninferior to HAS-BLED together with some other scoring systems [8]. Unlike our study, in addition to the presence of patients using NOAC in this study, patients with valvular AF were not included and CRNM bleeding was also included in the analysis. We believe that these differences may have affected the results. Considering that our results are more specific, especially in patients with intracranial hemorrhage, we think that a different perspective is presented in our study.

In a meta-analysis evaluating bleeding risk scoring, HAS-BLED scoring was evaluated as balanced in terms of specificity and sensitivity, while ORBIT, ATRIA and GARFIELD-AF scoring were found to have relatively high specificity but low sensitivity [11]. In the study by Burgess et al. the predictive power of bleeding risk scoring including HAS-BLED and ATRIA scores was expressed as weak [26]. Another meta-analysis evaluated the bleeding predictive power of HAS-BLED, ORBIT and ATRIA scoring rated as similar in patients using NOAC [27]. In a study analyzing 3550 patients on warfarin, both scoring systems showed a moderate predictive value for major bleeding. However, it was concluded that GARFIELD-AF scoring did not significantly improve the prediction of major and CRNM bleeding compared to HAS-BLED. In this study in which bleeding was classified as major, CRNM and any bleeding, HAS-BLED scoring was found superior to GARFIELD-AF scoring in the any bleeding group [9]. However, although there was a trend in favor of the GARFIELD-AF risk score in terms of predicting major bleeding in the high-risk group in this study, it did not reach statistical significance. In another study, evaluating patients using VKA and NOAC, Chichareon P et al. concluded that the major bleeding predictive values of HAS-BLED and GARFIELD-AF risk scoring were similar [28].

As can be seen at this point, although there is a tendency towards HAS-BLED in the literature, it seems that it would not be correct to talk about a clear superiority. In our study, we found that the GARFIELD-AF risk score has a better predictive power for major bleeding. It gives the impression that this scoring system may find a place in daily practice with its comprehensive and easily applicable parameters that evaluate stroke and mortality.

We think that our finding that the GARFIELD-AF risk score has a higher predictive value for major bleeding than an important scoring system such as HAS-BLED is important. However, as far as we know, no clear cut-off value has been established in which value ranges the risk is more pronounced. For example, it is known that the HAS-BLED score reaches a significant predictive value, especially when it is high [17,29]. In our study, the predictive power of the GARFIELD-AF risk score for major bleeding risk was found to be higher at values of 3.2% and above compared to other scores.

It is possible to put two main headings on treatment and follow-up in the patients using VKA. These are, of course, INR and TTR. In most cases, values between 2 and 3 are considered optimal, whereas slightly higher values such as 2.5–3.5 are accepted, especially in patients with mechanical mitral valves [30].

TTR rates can be monitored with a wide margin in different geographies. While values such as 68% have been observed in some studies [24,31], lower values such as 49% and 56% have been found especially in studies evaluating real life data [32,33]. Predicting the course of bleeding risk according to TTR rates, which are affected by both high and low INR, seems to be an even more complex calculation. For example, in a study evaluating major, CRNM and minor bleeding, TTR rates were found to be 43.1%, 49.3% and 51.3%, respectively [31]. This result can be interpreted as TTR was mostly outside the therapeutic range in the high INR direction. However, another study showed that the Asian patient group with lower INR levels and TTR rates had higher bleeding rates [33]. In our study, high INR and low TTR values were found to be significant in the patient group with major bleeding.

Among major hemorrhages, there is no doubt that intracranial hemorrhages are of particular importance. In a meta-analysis including 38 observational studies and evaluating more than one million patients, it was reported as 0.9% for VKA [16]. Similar levels are also observed in NOAC comparing warfarin studies [14,15]. In the study by Shen A.Y.J et al., very significant differences were found between races in terms of intracranial hemorrhage. The risk of intracranial hemorrhage was found to be 4 times higher in Asian patients using VKA and 2 times higher in Hispanic and black patients [34]. In the same study, intracranial hemorrhage patients were evaluated according to INR levels and no statistical difference was found between INR >3 or INR>4 groups. Huang et al. showed that high INR levels were associated with increased bleeding. Especially INR levels of 3 and above have been observed to be associated with bleeding [35]. The possibility that a complication such as intracranial hemorrhage waiting around the corner in VKA treatment, where metabolic interactions and thus individual differences may be very prominent, may be a source of justified distrust in clinicians, and the emphasis on NOAC in current guidelines may be recalled at this point [4,36]. As we have shown in our study, intracranial hemorrhages constitute 15.8% of major hemorrhages. These bleedings can be seen independently of INR and TTR levels.

When the demographic data of our study were analyzed, it was observed that advanced age, hypertension, impaired liver and kidney function, labil INR, antiaggregant use, malignancy, history of major and minor bleeding were risk factors for bleeding. The majority of these features are found in different bleeding risk scoring systems. However, a history of minor bleeding in the last year, which is not included in the scoring systems, is noteworthy as a feature with predictive value for major bleeding in our study.

This issue was questioned in the literature and it was reported that minor bleeding was not associated with increased major bleeding in the patient group using OAC [37]. The increased awareness of patients with minor bleeding may have been determinant. In fact, in another study on minor bleeding, it was shown that patients who had minor bleeding and continued their medication with a higher level of awareness about AF and anticoagulant treatment [38]. Another analysis from the ORBIT-AF patient population showed that bleeding events accounted for 20.1% of the reasons for discontinuation of treatment while using VKA [39]. In an analysis of GARFIELD-AF patients, those with minor bleeding had an increased mortality rate compared to patients with no bleeding. Although deaths in the minor bleeding group were attributed to bleeding at a lower rate, in the same study, 5.1% of 1098 patients with minor bleeding died after bleeding. Another remarkable statistical result is that when all-cause mortality was analyzed, it was 4% in patients who did not have bleeding and 5.3% in the minor bleeding group [31]. The importance of minor bleeding in patients on OAC and its impact on treatment dynamics seems to be less clear. In our study, patients with minor bleeding had a predictive value for major bleeding. At this point, although it puts a question mark on the innocence of minor bleeding, we believe that it may be a clear warning in terms of the need for better evaluation of dynamically changing modifiable bleeding risk factors rather than its effect on the course of treatment in clinical practice.

## 5 Conclusion

Atrial fibrillation is a private medical condition, both in terms of its pathophysiology and its treatment and the side effects that this treatment can cause. To this end, a good hemorrhage prediction can present the clinician an important advantage when balancing risk between stroke prevention and hemorrhage. Among the bleeding risk scores, the GARFIELD-AF risk score was superior to important bleeding scores such as HAS-BLED, ORBIT and ATRIA in terms of predicting major bleeding. Again, it is an important result that intracranial hemorrhages, which have a special place among major hemorrhages, were independent of INR and TTR levels. It is noteworthy that 8.2% of patients with major bleeding had a history of minor bleeding in the last year. It is also clear that larger scale studies are needed to make a more precise assessment.

## Author contribution statement

Sinan Yildirim: Conceived and designed the experiments; Performed the experiments; Analyzed and interpreted the data; Contributed reagents, materials, analysis tools or data; Wrote the paper.

Onur Aslan: Conceived and designed the experiments; Analyzed and interpreted the data; Contributed reagents, materials, analysis tools or data.

## Data availability statement

Data included in article/supplementary material/referenced in article.

## Declaration of competing interest

The authors declare that they have no known competing financial interests or personal relationships that could have appeared to influence the work reported in this paper.
